# Spotlight on the Uncommon: A Rare Case of a Malignant Glomus Tumor of the Skin

**DOI:** 10.7759/cureus.68524

**Published:** 2024-09-03

**Authors:** Claudia G Reid, Ashlyn S Everett

**Affiliations:** 1 Medicine, Edward Via College of Osteopathic Medicine (VCOM-Auburn), Auburn, USA; 2 Radiation Oncology, Edward Via College of Osteopathic Medicine (VCOM-Auburn), Auburn, USA

**Keywords:** glomus tumor of the skin, pathology, oncology, dermatology, malignant glomus tumor

## Abstract

Glomus tumors arise from a neuromyoarterial plexus origin, typically demonstrating branching vascular channels and aggregates of specialized glomus cells. They are characteristically identified in the subungual region of the hand, with the presentation of firm, red or blue nodules that are painful and sensitive to temperature. However, very few cases of malignant glomus tumors of the skin have been reported in the medical literature, making this case quite unique. This case report aims to identify an additional case of this rare condition, discuss the treatment plan pursued, and bring awareness to this distinct oncological entity.

A 41-year-old female patient initially presented to her primary care physician with an enlarging and painful nodule on her left temple that had been present for the last two months. The patient was then referred to plastic surgery for excision and pathological workup of the mass. The pathology report of the excised specimen revealed a malignant glomus tumor of the skin. The patient was subsequently scheduled for an oncologic workup with medical oncology and radiation oncology, and a wide excision was performed after the pathologic diagnosis. With negative margins from the wide excision and no evidence of disease spread, it was determined that there was no role for systemic therapy or radiation therapy at that time. Biannual dermatologic examination and monitoring are indicated for follow-up.

With very few reports of similar cases and little information in the medical literature on the treatment of this type of neoplasm, this is a unique and rare case that warrants discussion and exposure. Making the correct diagnosis, performing a complete workup, and removing the malignant glomus tumor are all essential parts of medical management in this case, and this type of neoplasm should not be excluded when evaluating the presentation of unusual cutaneous lesions.

## Introduction

Glomus tumors, typically identified in the subungual region of the distal extremities, have been postulated to be derived from modified smooth muscle cells of a neuromyoarterial glomus body, which is associated with thermoregulation [1]. Clinical characteristics of a glomus tumor can include severe pain and temperature-related algesia [2]. They most often present as well-defined blue or red nodules [3].

Most glomus tumors have been reported as benign neoplasms. However, there have been a few recorded cases of malignant glomus tumors in the medical literature, including cases presenting in the gastric fundus and the breast [4-6]. Benign glomus tumors typically show little, if any, mitotic activity. Glomus tumors are suspected to exhibit malignant potential when histologic features include nuclear atypia, increased mitotic activity (greater than 5 per 50 high-power fields (HPFs)), deeper location, infiltrative growth patterns, multicentricity, and size over 2 cm [7]. Glomus tumors typically demonstrate positive staining for smooth muscle actin [1], and they typically stain negative for desmin and human melanoma black 45 (HMB45) [1,8]. The Mayo Clinic performed a retrospective review from 1985 to 2005 to review extradigital glomus tumors to understand the clinical presentation better and establish guidelines for diagnosis and treatment [9]. Several different locations for extradigital glomus tumors were recognized. However, no case was identified with the neoplasm presenting on the temple, which continues to demonstrate the rarity and uniqueness of this case [9].

There is a separate tumor with a similar nomenclature, glomus jugulare, that should be differentiated from the malignant glomus tumor of the skin described in this case. Glomus jugulare is of paraganglioma origin and is typically located in the head and neck, as it arises within the jugular foramen and localizes to the jugular fossa. Paragangliomas are benign derivatives of neural crest cells, specifically the paraganglia [10]. Treatment of glomus jugulare has typically involved consideration of surgical resection and radiation therapy [11]. This distinction should be noted due to the separate nature of the pathophysiology of the two tumors and the differing treatment paradigms.

Although the literature is limited on malignant glomus tumors of the skin, wide excision after pathologic diagnosis appears to be sufficient initial management, with continuous follow-up [3,12]. Given the rarity of this case and the limited knowledge, this diagnosis should be considered when evaluating unusual skin lesions, as it can resemble a benign cyst, vascular or melanocytic lesion, fibroma, or lipoma.

## Case presentation

A 41-year-old Caucasian female presented to her primary care provider concerned about a painful nodule on her left temple that she noticed was enlarging and migrating superficially. She first noticed this entity about two months prior to her presentation. She additionally reported throbbing pain in the lesion with exposure to heat or with exercise. She had never experienced anything similar and had not noticed any additional similar or suspicious lesions. She had no remarkable family history and no family history of any such lesions. A 5 mm, well-circumscribed subcutaneous cystic lesion on the left temple was noted on physical exam. It was mobile, without observable evidence of infection. She was referred to plastic surgery for evaluation and lesion excision by her primary care provider.

**Figure 1:**
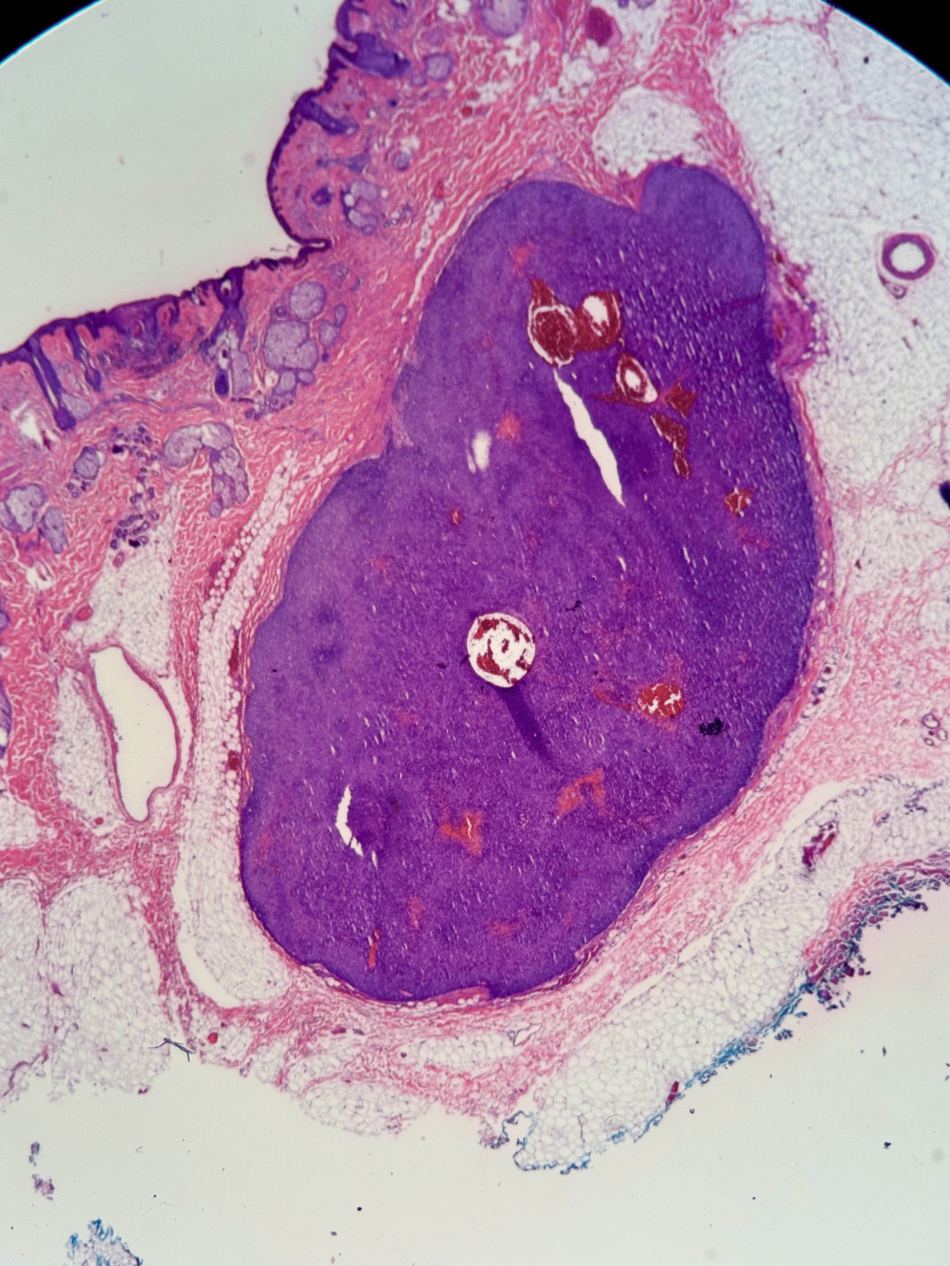
Low power image The skin surface can be observed at the 10:00-11:00 position. The tumor is represented by the dark central mass with deep margins at the 4:00-6:00 position.

**Figure 2:**
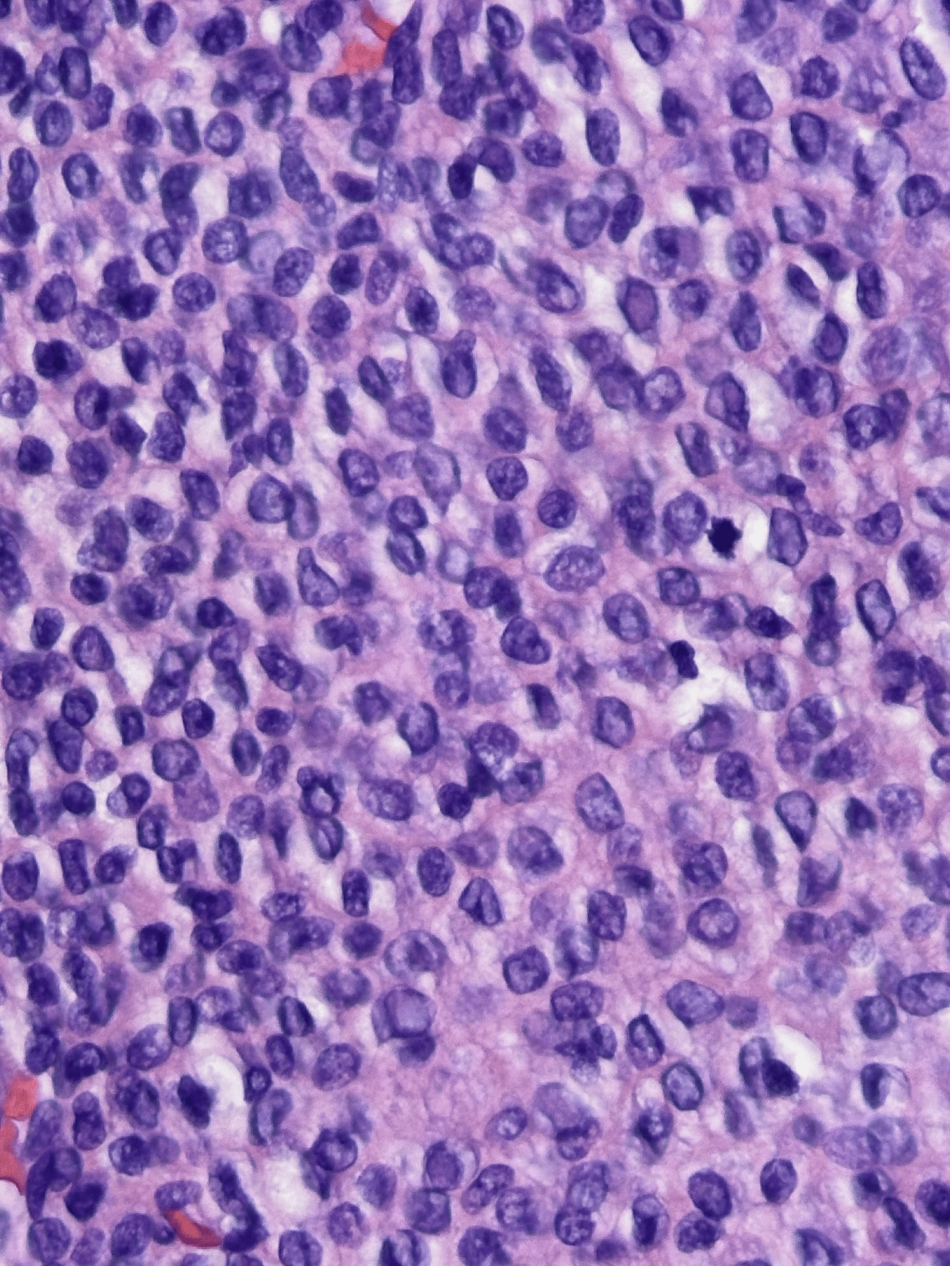
High power image This image demonstrates monomorphic tumor cells with mitotic figures.

**Figure 3:**
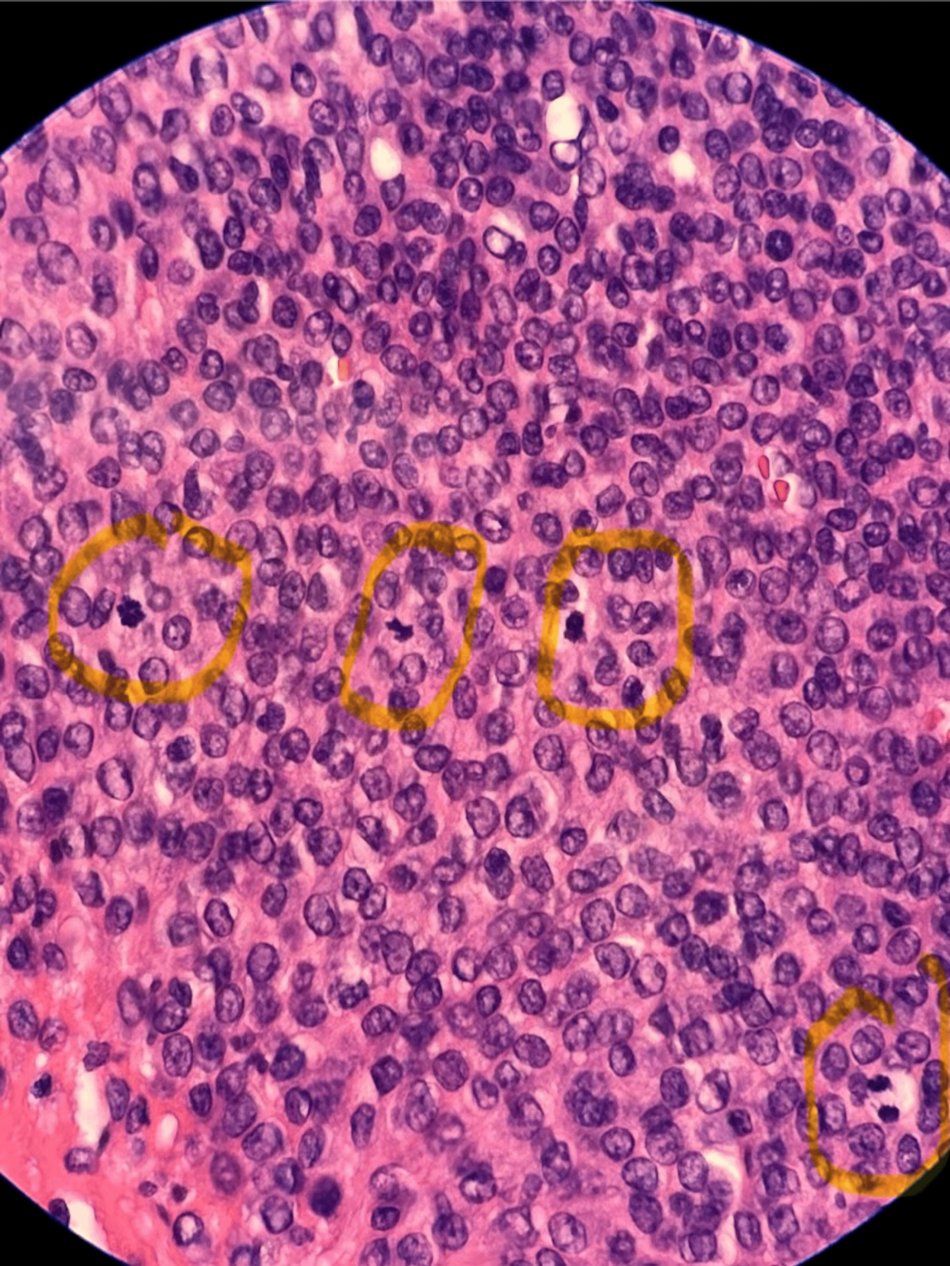
Mitotic figures present identified in histologic image In this image, greater than 10 mitotic figures per HPF are present. Some areas have 4 mitotic figures in a single HPF, which are circled in yellow by the pathologist. HPF: High power field

**Figure 4:**
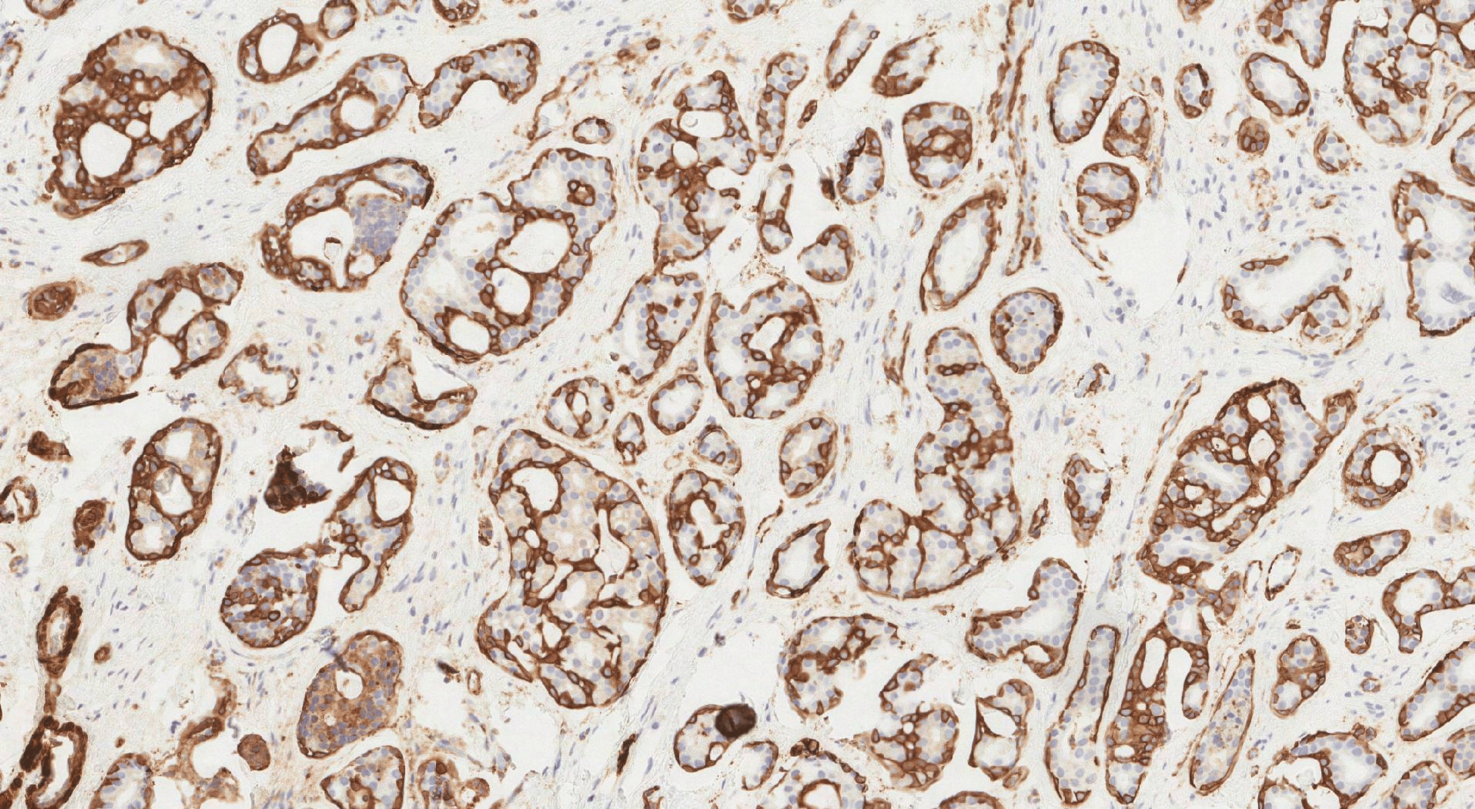
Smooth muscle actin-positive IHC stain This is an example of an image demonstrating smooth muscle actin-positive cells (in brown), observed by immunohistochemistry (IHC) [13].

Cyst excision was performed, and a 3-4 mm unencapsulated fatty growth was observed. It was then removed and sent for pathological evaluation. The initial surgical pathology report demonstrated a basaloid neoplasm with atypia. The pathologist described the specimen as having “sections demonstrating a proliferation of basaloid cells with associated vascular spaces,” as shown in Figures 1-2. There were increased mitotic figures and areas of suspicious necrosis as well. Figure 3 demonstrates over 10 mitotic figures per 10 HPFs, with some areas having four mitotic figures in a single HPF. A panel of immunohistochemical stains was performed to further evaluate this unusual case. The atypical cells demonstrated expression of smooth muscle actin (Figure 4) and were negative for p40, pancytokeratin, transcription factor SOX10, epithelial membrane antigen (EMA), progesterone receptor (PR), HMB45, and desmin. With the presence of mitotic figures and suspicious areas of necrosis, an aggressive neoplasm was suspected. The specimen was sent for further outside pathologic consultation. The final pathology report revealed that the cyst was consistent with a malignant glomus tumor of the skin.

**Figure 5:**
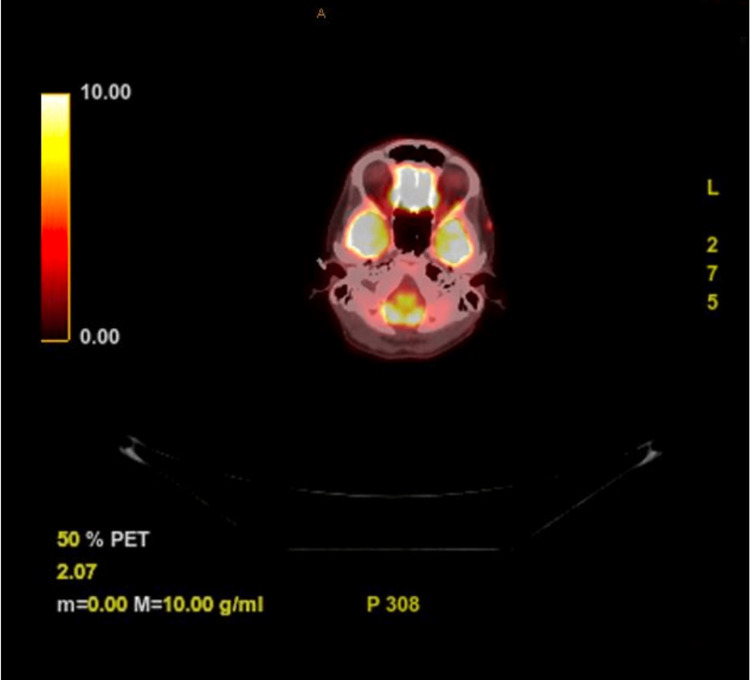
Axial view through the temple of the PET scan Small focus of minimal activity present in the left temporal region. PET: Positron emission tomography

**Figure 6:**
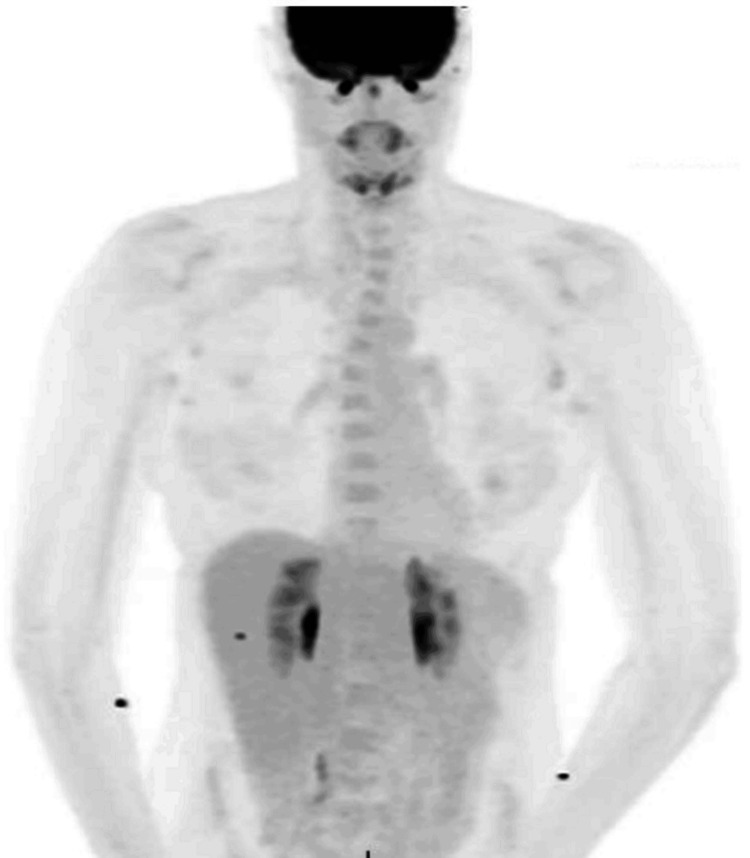
Maximum intensity projection (MIP) image of the PET scan Foci of activity on the skin forearms present bilaterally, likely due to contamination. PET: Positron emission tomography

After this diagnosis, the patient was referred for an oncologic workup due to the nature and uncertainty of this malignancy. She first met with medical oncology, where it was determined that there was no role for systemic therapy at that time, but she was sent for a positron emission tomography (PET) scan to rule out distant spread. The PET scan revealed a small focus of minimal activity in the left temporal region and foci of activity on the skin of the forearms bilaterally, likely due to contamination (Figures 5-6).

She then presented to radiation oncology for treatment recommendations. Due to the glomus tumor of the skin being a distinct entity from glomus jugulare, which is commonly treated with radiation therapy, it was determined that radiation therapy is also not indicated at this time, pending negative margins on wide excision.

**Figure 7:**
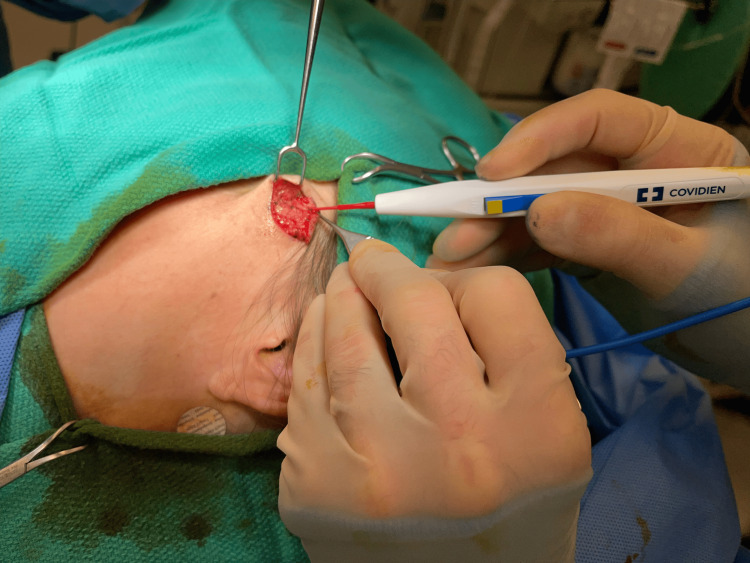
Wide excision of glomus tumor

**Figure 8:**
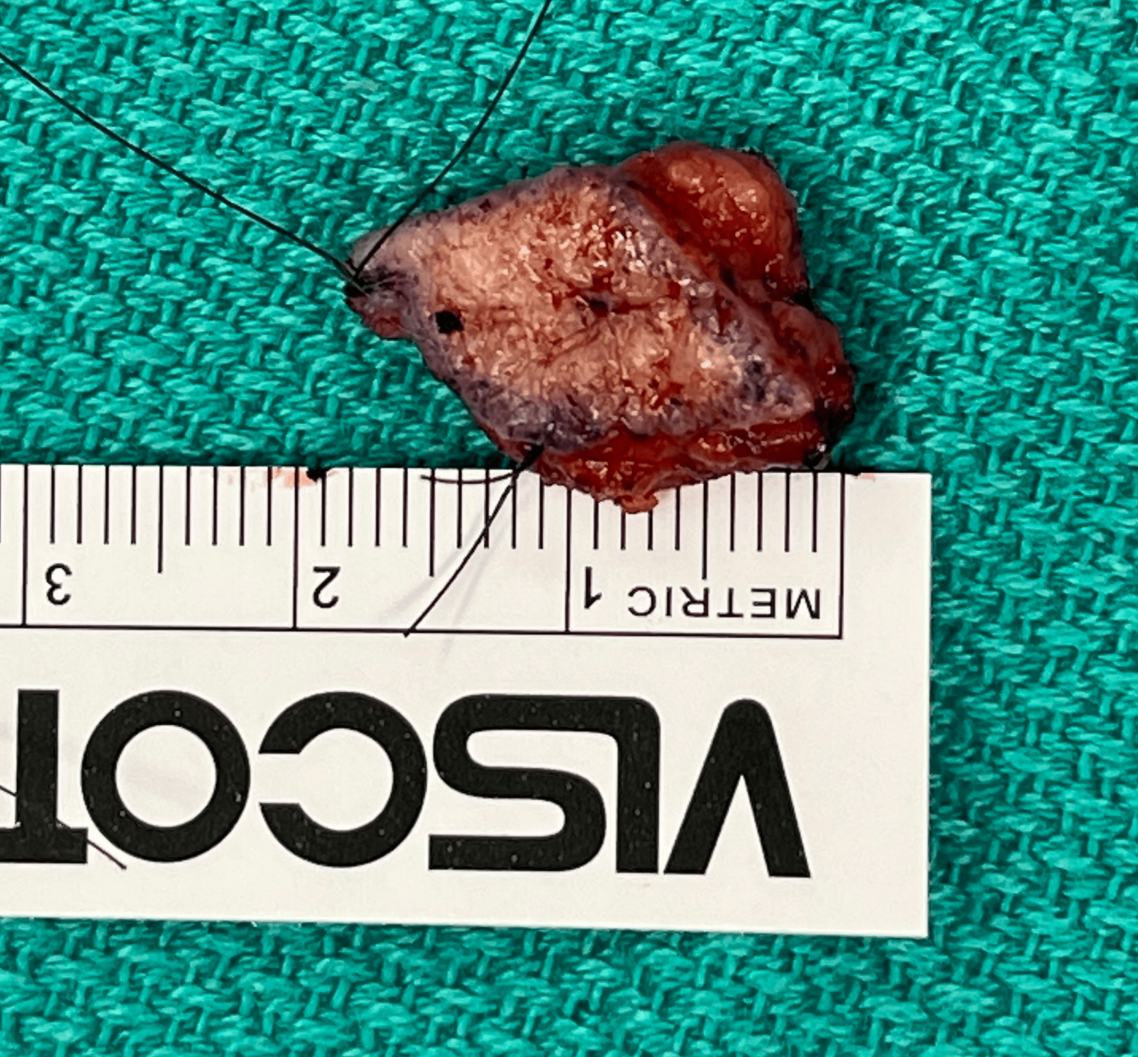
Resected glomus tumor measuring 2 x 1 cm

**Figure 9:**
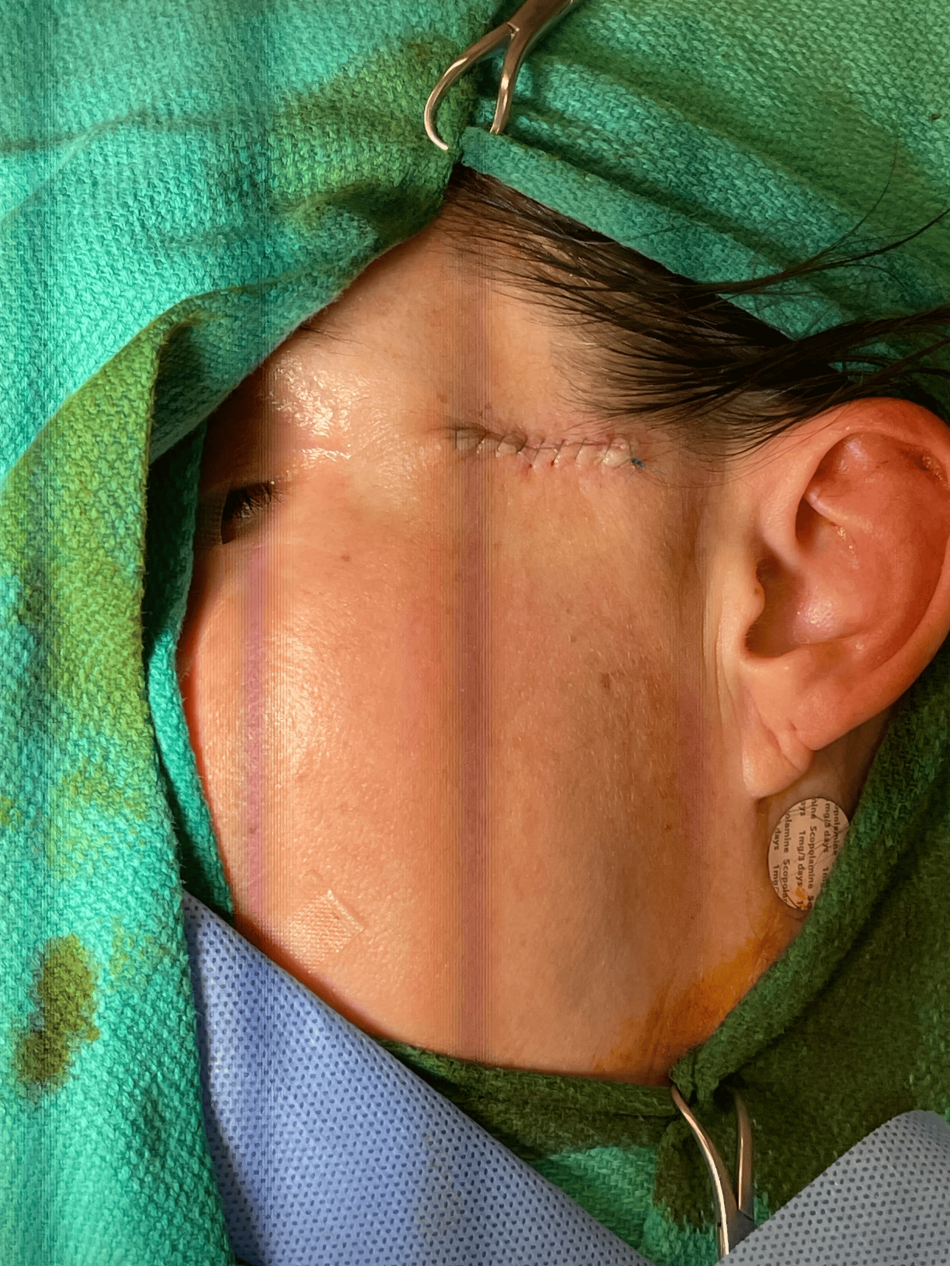
Post-excision presentation

After research and discussion, it was determined that wide local excision with negative margins is the appropriate initial, and hopefully definitive, treatment. The patient underwent wide excision with plastic surgery, and the tumor measured 2 x 1 cm, as demonstrated in Figures 7-9. The surgical pathology report demonstrated a malignant glomus tumor present in the subcutis, with negative margins and without angiolymphatic or perineural invasion. The lesion was described as well-circumscribed and was approximately 1 mm away from the central deep margin.

After the wide excision, the patient was encouraged to follow up with dermatology every six months for regular skin checks and monitoring. A discussion about observing for adenopathy in the head and neck was conducted, as this would likely be the next station where the disease could spread. Verbal informed consent was obtained from the patient for the case report and publication.

## Discussion

In the case described, this patient was diagnosed with a malignant glomus tumor of the skin after excisional biopsy and pathologic analysis. On examination, the patient presented with an enlarging mass on her left temple that was extremely painful and sensitive to heat. These presenting symptoms are characteristic of glomus tumors. However, the lesion was in an unusual location for a glomus tumor, as they are typically present in the subungual region or distal extremities [1]. Given the unlikely location and distinguishing presentation, this is a unique case to cite, as fewer than 50 reports of cutaneous malignant glomus tumors have been recorded [14].

Glomus tumors represent 1-2% of all soft tissue tumors, with the majority being classified as benign [3]. Malignant glomus tumors have been determined to develop de novo or from a prior benign tumor, which is very rare [14]. Glomus bodies are found in the reticular dermis and are contractile neuromyoarterial structures. They are a specialized form of arteriovenous anastomosis, and the glomus body assists with blood flow within the skin. It can affect thermoregulation, causing the clinical finding of sensitivity to temperature change [1]. Glomus tumors can be exceptionally painful, with throbbing or lancinating discomfort and severe pain out of proportion to minor trauma [15]. With the bothersome and concerning symptoms that usually accompany glomus tumors, a quest for the correct diagnosis, followed by proper treatment, should be pursued without delay.

Potential differential diagnoses may include dermatologic entities such as a benign cyst, vascular or melanocytic lesion, fibroma, or lipoma. Due to the unusual location and presenting symptoms, an excisional biopsy with pathologic examination was warranted to help establish a definitive diagnosis and avoid misdiagnosis. This is a rare neoplasm that should receive a well-rounded approach to management from several specialties: plastic surgery to perform the excision; medical oncology for systemic treatment recommendations and assistance in coordinating oncologic follow-up care; radiation oncology for local treatment recommendations and continued assistance in coordinating oncologic follow-up care; and dermatology for regular, comprehensive physical examination follow-up.

Surgical resection with wide excision is a widely accepted treatment method for glomus tumors, although most literature reports focus on the benign type [3]. Incomplete resection should be avoided, as it can increase the risk of recurrence [16]. The treating physicians utilized the limited available literature, along with their clinical knowledge and experience treating similar cases and presentations, to make their respective recommendations for treatment - leading to the plan of wide excision with negative margins, without systemic or radiation therapy, and with regular dermatology follow-up.

## Conclusions

Although rare, and with few similar clinical reports, malignant glomus tumors of the skin should not be excluded when examining unusual lesions of the skin. With the treatment plan of wide excision with negative margins and close, regular dermatology follow-up, it is hoped that recurrence and metastasis will not occur, and that this treatment will have been definitive for this patient.
